# 3-[4-Amino-3-(4-methyl­phen­yl)-5-sulfanyl­idene-4,5-dihydro-1*H*-1,2,4-triazol-1-yl]-3-(2-chloro­phen­yl)-1-phenyl­propan-1-one

**DOI:** 10.1107/S1600536811023993

**Published:** 2011-06-25

**Authors:** Wei Wang, Qing-lei Liu, Xiao-yu Jia, Jing-jing Zhang, Yan Gao

**Affiliations:** a School of Perfume and Aroma Technology, Shanghai Institute of Technology, Shanghai 200235, People’s Republic of China; b School of Chemical Engineering, University of Science and Technology LiaoNing, Anshan 114051, People’s Republic of China

## Abstract

In the title mol­ecule, C_24_H_21_ClN_4_OS, the 1,2,4-triazole ring forms dihedral angles of 37.2 (2), 71.9 (2) and 84.9 (2) ° with the three benzene rings. In the crystal, weak inter­molecular N—H⋯S hydrogen bonds link the mol­ecules into centrosymmetric dimers.

## Related literature

For the crystal structures of related 1,2,4-triazole-5(4*H*)-thione derivatives, see: Al-Tamimi *et al.* (2010 ▶); Fun *et al.* (2009 ▶); Tan *et al.* (2010 ▶); Wang *et al.* (2011 ▶).
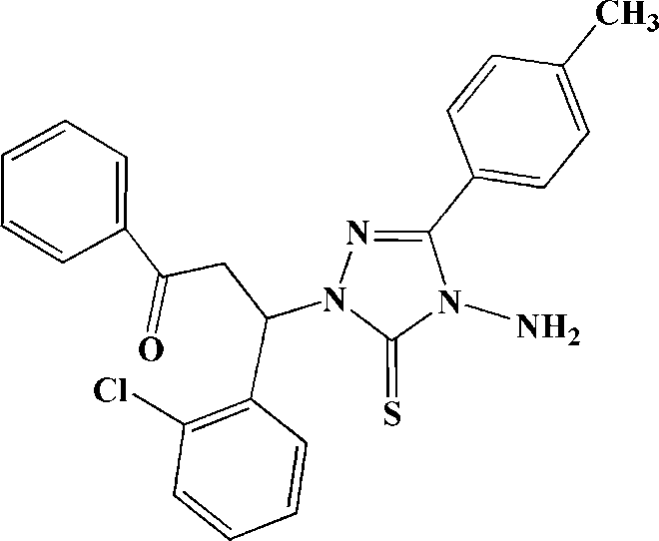


## Experimental

### 

#### Crystal data

C_24_H_21_ClN_4_OS
*M* *_r_* = 448.96
Monoclinic, 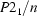

*a* = 10.9873 (12) Å
*b* = 11.8220 (14) Å
*c* = 17.438 (3) Å
β = 94.828 (7)°
*V* = 2257.1 (5) Å^3^
*Z* = 4
Mo *K*α radiation
μ = 0.29 mm^−1^
*T* = 113 K
0.30 × 0.08 × 0.08 mm

#### Data collection

Rigaku Saturn CCD area-detector diffractometer
Absorption correction: multi-scan (*CrystalClear*; Rigaku/MSC, 2005 ▶) *T*_min_ = 0.919, *T*_max_ = 0.978
22007 measured reflections
4989 independent reflections
3972 reflections with *I* > 2σ(*I*)
*R*_int_ = 0.065

#### Refinement

*R*[*F* ^2^ > 2σ(*F* ^2^)] = 0.060
*wR*(*F* ^2^) = 0.145
*S* = 1.11
4989 reflections
289 parameters
H atoms treated by a mixture of independent and constrained refinement
Δρ_max_ = 0.93 e Å^−3^
Δρ_min_ = −0.76 e Å^−3^

### 

Data collection: *CrystalClear* (Rigaku/MSC, 2005 ▶); cell refinement: *CrystalClear*; data reduction: *CrystalClear*; program(s) used to solve structure: *SHELXS97* (Sheldrick, 2008 ▶); program(s) used to refine structure: *SHELXL97* (Sheldrick, 2008 ▶); molecular graphics: *SHELXTL* (Sheldrick, 2008 ▶); software used to prepare material for publication: *SHELXTL*.

## Supplementary Material

Crystal structure: contains datablock(s) global, I. DOI: 10.1107/S1600536811023993/cv5109sup1.cif

Structure factors: contains datablock(s) I. DOI: 10.1107/S1600536811023993/cv5109Isup2.hkl

Supplementary material file. DOI: 10.1107/S1600536811023993/cv5109Isup3.cml

Additional supplementary materials:  crystallographic information; 3D view; checkCIF report

## Figures and Tables

**Table 1:** Hydrogen-bond geometry (Å, °)

*D*—H⋯*A*	*D*—H	H⋯*A*	*D*⋯*A*	*D*—H⋯*A*
N4—H4*B*⋯S1^i^	0.93 (3)	2.77 (3)	3.526 (3)	139 (2)

Symmetry code: (i) 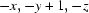.

**Fig. 1.**
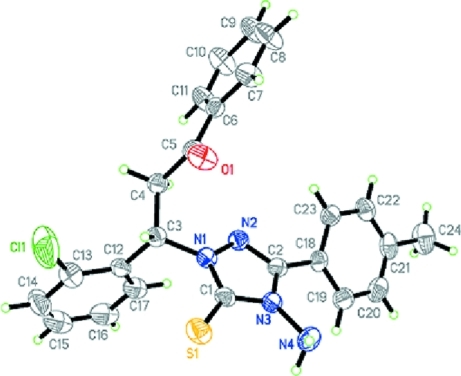
View of the molecule of (I) showing the atom-labelling scheme. Displacement ellipsoids are drawn at the 55% probability level.

## supplementary crystallographic information

### Comment

In continuation of structural study of 1,2,4-triazole-5(4*H*)-thione derivatives in our group (Wang *et al.*, 2011), we present here the crystal structure of the title compound, (I).

In (I) (Fig.1), all bond lengths and angles are normal and comparable with those observed in related structures (Al-Tamimi *et al.*, 2010; Fun *et al.*, 2009; Tan *et al.*, 2010; Wang *et al.*, 2011). The C1 atom in the triazole ring deviates from the normal C*_sp_*^2^ hybridization state having the bond angles of 102.49 (19)° (N1—C1—N3) and 129.67 (18)° (N1—C1—S1). The three benzene rings in the molecule are inclined with respect to the 1,2,4-triazole ring [dihedral angles of 37.7 (2)° (C18—C23), 71.9 (2)° (C6—C11) and 84.9 (2)% (C12—C17)]. Benzene ring A (C18—C23) attached to the triazole ring makes the dihedral angles of 97.3 (2) and 84.7 (2)° with the benzene rings B (C6—C11) and C (C12—C17), respectively. Rings B and C form a dihedral angle of 60.4 (2)°.

In the crystal structure, intermolecular N—H···S hydrogen bonds (Table 1) link the adjacent molecules into centrosymmetric dimers.

### Experimental

The title compound was synthesized by the reaction of the 3-(2-chlorophenyl)-1- (4-methylphenyl)-2-propen-1-one (2.0 mmol) with 4-amino-3-phenyl-4*H*-1,2,4- triazole-5-thiol (2.0 mmol) in ethanol. The reaction progress was monitored *via* TLC. The resulting precipitate was filtered off, washed with cold ethanol, dried and purified to give the target product as colourless solid in 75% yield. Crystals of (I) suitable for single-crystal X-ray analysis were grown by slow evaporation of a solution in chloroform-ethanol (1:1).

### Refinement

The H atoms attached to N atoms were located on a difference map and isotropically refined. C-bound H atoms were positioned geometrically (C—H = 0.95–1.00 Å)  and refined as riding, with *U*_iso_(H) = 1.2-1.5*U*_eq_(C).

### Crystal data

**Table:**

C_24_H_21_ClN_4_OS	*F*(000) = 936
*M**_r_* = 448.96	*D*_x_ = 1.321 Mg m^−^^3^
Monoclinic, *P*2_1_/*n*	Mo *K*α radiation, λ = 0.71073 Å
Hall symbol: -P 2yn	Cell parameters from 6624 reflections
*a* = 10.9873 (12) Å	θ = 1.7–27.2°
*b* = 11.8220 (14) Å	µ = 0.29 mm^−^^1^
*c* = 17.438 (3) Å	*T* = 113 K
β = 94.828 (7)°	Prism, colourless
*V* = 2257.1 (5) Å^3^	0.30 × 0.08 × 0.08 mm
*Z* = 4	

### Data collection

**Table:**

Rigaku Saturn CCD area-detector diffractometer	4989 independent reflections
Radiation source: rotating anode	3972 reflections with *I* > 2σ(*I*)
multilayer	*R*_int_ = 0.065
Detector resolution: 14.63 pixels mm^-1^	θ_max_ = 27.2°, θ_min_ = 2.1°
φ and ω scans	*h* = −14→14
Absorption correction: multi-scan (*CrystalClear*; Rigaku/MSC, 2005)	*k* = −15→15
*T*_min_ = 0.919, *T*_max_ = 0.978	*l* = −20→22
22007 measured reflections	

### Refinement

**Table:**

Refinement on *F*^2^	Primary atom site location: structure-invariant direct methods
Least-squares matrix: full	Secondary atom site location: difference Fourier map
*R*[*F*^2^ > 2σ(*F*^2^)] = 0.060	Hydrogen site location: inferred from neighbouring sites
*wR*(*F*^2^) = 0.145	H atoms treated by a mixture of independent and constrained refinement
*S* = 1.11	*w* = 1/[σ^2^(*F*_o_^2^) + (0.058*P*)^2^ + 0.3265*P*] where *P* = (*F*_o_^2^ + 2*F*_c_^2^)/3
4989 reflections	(Δ/σ)_max_ = 0.002
289 parameters	Δρ_max_ = 0.93 e Å^−^^3^
0 restraints	Δρ_min_ = −0.76 e Å^−^^3^

### Special details

**Table:**

Geometry. All e.s.d.'s (except the e.s.d. in the dihedral angle between two l.s. planes) are estimated using the full covariance matrix. The cell e.s.d.'s are taken into account individually in the estimation of e.s.d.'s in distances, angles and torsion angles; correlations between e.s.d.'s in cell parameters are only used when they are defined by crystal symmetry. An approximate (isotropic) treatment of cell e.s.d.'s is used for estimating e.s.d.'s involving l.s. planes.
Refinement. Refinement of *F*^2^ against ALL reflections. The weighted *R*-factor *wR* and goodness of fit *S* are based on *F*^2^, conventional *R*-factors *R* are based on *F*, with *F* set to zero for negative *F*^2^. The threshold expression of *F*^2^ > σ(*F*^2^) is used only for calculating *R*-factors(gt) *etc*. and is not relevant to the choice of reflections for refinement. *R*-factors based on *F*^2^ are statistically about twice as large as those based on *F*, and *R*- factors based on ALL data will be even larger.

### Fractional atomic coordinates and isotropic or equivalent isotropic displacement parameters (Å^2^)

**Table:**

	*x*	*y*	*z*	*U*_iso_*/*U*_eq_	
S1	0.02423 (6)	0.49557 (5)	0.11191 (4)	0.03397 (19)	
Cl1	−0.32308 (8)	0.64505 (9)	0.19347 (5)	0.0734 (3)	
O1	0.05692 (16)	0.81675 (14)	0.24353 (10)	0.0345 (4)	
N1	0.00694 (16)	0.72168 (16)	0.08127 (10)	0.0235 (4)	
N2	0.06436 (17)	0.80772 (15)	0.04491 (11)	0.0248 (4)	
N3	0.15533 (16)	0.64405 (16)	0.03124 (11)	0.0260 (4)	
N4	0.2416 (2)	0.5653 (2)	0.00864 (18)	0.0378 (6)	
C1	0.0594 (2)	0.61979 (19)	0.07433 (13)	0.0253 (5)	
C2	0.15630 (19)	0.75776 (19)	0.01521 (13)	0.0234 (5)	
C3	−0.1037 (2)	0.7430 (2)	0.12081 (13)	0.0254 (5)	
H3	−0.1025	0.6921	0.1667	0.030*	
C4	−0.1022 (2)	0.86520 (19)	0.14850 (13)	0.0265 (5)	
H4C	−0.1026	0.9162	0.1034	0.032*	
H4D	−0.1772	0.8801	0.1745	0.032*	
C5	0.0087 (2)	0.8918 (2)	0.20371 (13)	0.0248 (5)	
C6	0.05468 (19)	1.01009 (19)	0.20922 (12)	0.0238 (5)	
C7	0.1565 (2)	1.0353 (2)	0.25923 (15)	0.0358 (6)	
H7	0.1963	0.9764	0.2888	0.043*	
C8	0.2006 (3)	1.1444 (2)	0.26661 (16)	0.0448 (7)	
H8	0.2718	1.1598	0.2998	0.054*	
C9	0.1412 (2)	1.2315 (2)	0.22572 (16)	0.0422 (7)	
H9	0.1695	1.3071	0.2320	0.051*	
C10	0.0406 (2)	1.2074 (2)	0.17581 (16)	0.0412 (7)	
H10	−0.0002	1.2667	0.1473	0.049*	
C11	−0.0015 (2)	1.0977 (2)	0.16691 (14)	0.0321 (6)	
H11	−0.0698	1.0821	0.1314	0.038*	
C12	−0.2167 (2)	0.7165 (2)	0.06798 (14)	0.0276 (5)	
C13	−0.3211 (2)	0.6709 (2)	0.09597 (16)	0.0413 (7)	
C14	−0.4247 (2)	0.6455 (2)	0.0482 (2)	0.0495 (8)	
H14	−0.4943	0.6136	0.0689	0.059*	
C15	−0.4263 (3)	0.6667 (2)	−0.02916 (19)	0.0473 (8)	
H15	−0.4967	0.6487	−0.0623	0.057*	
C16	−0.3258 (3)	0.7140 (2)	−0.05900 (16)	0.0416 (7)	
H16	−0.3274	0.7299	−0.1125	0.050*	
C17	−0.2220 (2)	0.7383 (2)	−0.01052 (15)	0.0337 (6)	
H17	−0.1530	0.7706	−0.0316	0.040*	
C18	0.24385 (19)	0.8188 (2)	−0.02880 (13)	0.0249 (5)	
C19	0.2891 (2)	0.7736 (2)	−0.09446 (14)	0.0320 (6)	
H19	0.2670	0.6993	−0.1110	0.038*	
C20	0.3669 (2)	0.8382 (2)	−0.13548 (14)	0.0361 (6)	
H20	0.3978	0.8068	−0.1801	0.043*	
C21	0.4010 (2)	0.9470 (2)	−0.11339 (14)	0.0345 (6)	
C22	0.3555 (2)	0.9906 (2)	−0.04791 (15)	0.0317 (6)	
H22	0.3780	1.0649	−0.0314	0.038*	
C23	0.2777 (2)	0.9280 (2)	−0.00594 (14)	0.0279 (5)	
H23	0.2472	0.9598	0.0387	0.033*	
C24	0.4854 (3)	1.0158 (3)	−0.15917 (17)	0.0521 (8)	
H24A	0.5002	1.0896	−0.1345	0.078*	
H24B	0.4476	1.0268	−0.2116	0.078*	
H24C	0.5631	0.9757	−0.1611	0.078*	
H4A	0.257 (3)	0.526 (3)	0.0465 (18)	0.046 (10)*	
H4B	0.201 (3)	0.518 (2)	−0.0274 (16)	0.040 (8)*	

### Atomic displacement parameters (Å^2^)

**Table:**

	*U*^11^	*U*^22^	*U*^33^	*U*^12^	*U*^13^	*U*^23^
S1	0.0322 (3)	0.0223 (4)	0.0465 (4)	−0.0008 (3)	−0.0020 (3)	0.0056 (3)
Cl1	0.0556 (5)	0.1144 (8)	0.0517 (5)	−0.0340 (5)	0.0137 (4)	0.0089 (5)
O1	0.0406 (10)	0.0255 (10)	0.0351 (10)	0.0017 (8)	−0.0099 (8)	0.0046 (8)
N1	0.0227 (10)	0.0214 (10)	0.0258 (10)	0.0006 (8)	−0.0008 (7)	0.0011 (8)
N2	0.0253 (10)	0.0208 (10)	0.0283 (11)	0.0008 (8)	0.0015 (8)	0.0007 (8)
N3	0.0202 (10)	0.0208 (11)	0.0364 (12)	0.0037 (8)	−0.0021 (8)	−0.0019 (9)
N4	0.0296 (12)	0.0249 (13)	0.0590 (17)	0.0091 (10)	0.0051 (11)	−0.0017 (12)
C1	0.0211 (11)	0.0233 (13)	0.0299 (13)	0.0008 (9)	−0.0067 (9)	−0.0037 (10)
C2	0.0222 (11)	0.0216 (12)	0.0254 (12)	0.0027 (9)	−0.0044 (9)	−0.0011 (9)
C3	0.0259 (12)	0.0256 (13)	0.0247 (12)	−0.0005 (10)	0.0023 (9)	−0.0007 (10)
C4	0.0246 (12)	0.0261 (13)	0.0284 (13)	0.0019 (10)	0.0000 (9)	−0.0029 (10)
C5	0.0246 (12)	0.0273 (13)	0.0226 (12)	0.0033 (10)	0.0017 (9)	−0.0028 (10)
C6	0.0225 (11)	0.0271 (13)	0.0221 (12)	0.0001 (9)	0.0033 (9)	0.0000 (9)
C7	0.0364 (14)	0.0276 (14)	0.0413 (15)	0.0038 (11)	−0.0102 (11)	0.0013 (12)
C8	0.0398 (16)	0.0377 (17)	0.0529 (18)	−0.0070 (13)	−0.0200 (13)	0.0024 (13)
C9	0.0423 (15)	0.0306 (15)	0.0521 (18)	−0.0129 (12)	−0.0046 (13)	0.0048 (13)
C10	0.0408 (15)	0.0325 (15)	0.0476 (17)	−0.0059 (12)	−0.0124 (12)	0.0124 (13)
C11	0.0292 (13)	0.0299 (14)	0.0352 (14)	−0.0032 (10)	−0.0088 (10)	0.0087 (11)
C12	0.0230 (12)	0.0246 (13)	0.0346 (14)	0.0003 (10)	−0.0006 (9)	−0.0075 (11)
C13	0.0343 (14)	0.0441 (17)	0.0453 (16)	−0.0057 (12)	0.0026 (12)	−0.0060 (13)
C14	0.0254 (14)	0.0420 (18)	0.080 (2)	−0.0056 (12)	−0.0003 (14)	−0.0081 (16)
C15	0.0326 (15)	0.0358 (16)	0.069 (2)	0.0100 (12)	−0.0210 (14)	−0.0123 (15)
C16	0.0424 (16)	0.0352 (16)	0.0441 (16)	0.0098 (12)	−0.0157 (12)	−0.0064 (13)
C17	0.0323 (13)	0.0302 (14)	0.0369 (15)	0.0044 (11)	−0.0067 (10)	−0.0023 (11)
C18	0.0188 (11)	0.0264 (13)	0.0290 (12)	0.0058 (9)	−0.0016 (9)	0.0037 (10)
C19	0.0290 (13)	0.0350 (15)	0.0313 (14)	0.0040 (11)	−0.0022 (10)	−0.0027 (11)
C20	0.0304 (13)	0.0516 (18)	0.0265 (13)	0.0094 (12)	0.0028 (10)	0.0013 (12)
C21	0.0227 (12)	0.0462 (17)	0.0339 (14)	0.0019 (11)	−0.0010 (10)	0.0110 (12)
C22	0.0270 (13)	0.0299 (14)	0.0377 (14)	−0.0024 (10)	−0.0001 (10)	0.0065 (11)
C23	0.0264 (12)	0.0278 (14)	0.0293 (13)	0.0043 (10)	0.0016 (9)	0.0013 (10)
C24	0.0441 (17)	0.071 (2)	0.0422 (18)	−0.0056 (15)	0.0100 (13)	0.0162 (15)

### Geometric parameters (Å, °)

**Table:**

S1—C1	1.667 (2)	C10—C11	1.381 (3)
Cl1—C13	1.730 (3)	C10—H10	0.9500
O1—C5	1.220 (3)	C11—H11	0.9500
N1—C1	1.345 (3)	C12—C17	1.390 (3)
N1—N2	1.379 (2)	C12—C13	1.391 (3)
N1—C3	1.468 (3)	C13—C14	1.387 (4)
N2—C2	1.314 (3)	C14—C15	1.371 (4)
N3—C2	1.373 (3)	C14—H14	0.9500
N3—C1	1.375 (3)	C15—C16	1.378 (4)
N3—N4	1.409 (3)	C15—H15	0.9500
N4—H4A	0.81 (3)	C16—C17	1.392 (3)
N4—H4B	0.93 (3)	C16—H16	0.9500
C2—C18	1.469 (3)	C17—H17	0.9500
C3—C12	1.516 (3)	C18—C23	1.393 (3)
C3—C4	1.523 (3)	C18—C19	1.393 (3)
C3—H3	1.0000	C19—C20	1.388 (3)
C4—C5	1.520 (3)	C19—H19	0.9500
C4—H4C	0.9900	C20—C21	1.385 (4)
C4—H4D	0.9900	C20—H20	0.9500
C5—C6	1.487 (3)	C21—C22	1.384 (3)
C6—C11	1.386 (3)	C21—C24	1.511 (4)
C6—C7	1.392 (3)	C22—C23	1.385 (3)
C7—C8	1.381 (4)	C22—H22	0.9500
C7—H7	0.9500	C23—H23	0.9500
C8—C9	1.384 (4)	C24—H24A	0.9800
C8—H8	0.9500	C24—H24B	0.9800
C9—C10	1.378 (4)	C24—H24C	0.9800
C9—H9	0.9500		
			
C1—N1—N2	113.77 (18)	C10—C11—C6	120.8 (2)
C1—N1—C3	124.99 (19)	C10—C11—H11	119.6
N2—N1—C3	121.21 (18)	C6—C11—H11	119.6
C2—N2—N1	104.26 (18)	C17—C12—C13	116.8 (2)
C2—N3—C1	109.56 (18)	C17—C12—C3	121.6 (2)
C2—N3—N4	124.99 (19)	C13—C12—C3	121.6 (2)
C1—N3—N4	125.4 (2)	C14—C13—C12	122.2 (3)
N3—N4—H4A	104 (2)	C14—C13—Cl1	118.3 (2)
N3—N4—H4B	107.0 (17)	C12—C13—Cl1	119.5 (2)
H4A—N4—H4B	105 (3)	C15—C14—C13	119.5 (3)
N1—C1—N3	102.49 (19)	C15—C14—H14	120.2
N1—C1—S1	129.67 (18)	C13—C14—H14	120.2
N3—C1—S1	127.75 (17)	C14—C15—C16	120.1 (3)
N2—C2—N3	109.91 (19)	C14—C15—H15	119.9
N2—C2—C18	123.0 (2)	C16—C15—H15	119.9
N3—C2—C18	127.1 (2)	C15—C16—C17	119.7 (3)
N1—C3—C12	110.34 (18)	C15—C16—H16	120.1
N1—C3—C4	108.91 (18)	C17—C16—H16	120.1
C12—C3—C4	112.02 (18)	C12—C17—C16	121.6 (3)
N1—C3—H3	108.5	C12—C17—H17	119.2
C12—C3—H3	108.5	C16—C17—H17	119.2
C4—C3—H3	108.5	C23—C18—C19	119.0 (2)
C5—C4—C3	112.57 (19)	C23—C18—C2	118.5 (2)
C5—C4—H4C	109.1	C19—C18—C2	122.4 (2)
C3—C4—H4C	109.1	C20—C19—C18	119.4 (2)
C5—C4—H4D	109.1	C20—C19—H19	120.3
C3—C4—H4D	109.1	C18—C19—H19	120.3
H4C—C4—H4D	107.8	C21—C20—C19	122.1 (2)
O1—C5—C6	121.2 (2)	C21—C20—H20	119.0
O1—C5—C4	119.8 (2)	C19—C20—H20	119.0
C6—C5—C4	119.02 (19)	C22—C21—C20	117.9 (2)
C11—C6—C7	118.2 (2)	C22—C21—C24	121.0 (3)
C11—C6—C5	122.3 (2)	C20—C21—C24	121.1 (2)
C7—C6—C5	119.5 (2)	C21—C22—C23	121.2 (2)
C8—C7—C6	121.0 (2)	C21—C22—H22	119.4
C8—C7—H7	119.5	C23—C22—H22	119.4
C6—C7—H7	119.5	C22—C23—C18	120.4 (2)
C7—C8—C9	120.0 (2)	C22—C23—H23	119.8
C7—C8—H8	120.0	C18—C23—H23	119.8
C9—C8—H8	120.0	C21—C24—H24A	109.5
C10—C9—C8	119.4 (3)	C21—C24—H24B	109.5
C10—C9—H9	120.3	H24A—C24—H24B	109.5
C8—C9—H9	120.3	C21—C24—H24C	109.5
C9—C10—C11	120.6 (2)	H24A—C24—H24C	109.5
C9—C10—H10	119.7	H24B—C24—H24C	109.5
C11—C10—H10	119.7		
			
C1—N1—N2—C2	−0.7 (2)	C9—C10—C11—C6	1.5 (4)
C3—N1—N2—C2	−178.69 (18)	C7—C6—C11—C10	−1.7 (4)
N2—N1—C1—N3	0.1 (2)	C5—C6—C11—C10	177.3 (2)
C3—N1—C1—N3	178.02 (19)	N1—C3—C12—C17	−36.0 (3)
N2—N1—C1—S1	176.98 (16)	C4—C3—C12—C17	85.5 (3)
C3—N1—C1—S1	−5.1 (3)	N1—C3—C12—C13	145.1 (2)
C2—N3—C1—N1	0.5 (2)	C4—C3—C12—C13	−93.4 (3)
N4—N3—C1—N1	178.9 (2)	C17—C12—C13—C14	1.6 (4)
C2—N3—C1—S1	−176.44 (17)	C3—C12—C13—C14	−179.5 (2)
N4—N3—C1—S1	2.0 (3)	C17—C12—C13—Cl1	−178.24 (19)
N1—N2—C2—N3	1.0 (2)	C3—C12—C13—Cl1	0.7 (3)
N1—N2—C2—C18	−179.51 (19)	C12—C13—C14—C15	−0.7 (4)
C1—N3—C2—N2	−1.0 (2)	Cl1—C13—C14—C15	179.1 (2)
N4—N3—C2—N2	−179.4 (2)	C13—C14—C15—C16	−0.7 (4)
C1—N3—C2—C18	179.5 (2)	C14—C15—C16—C17	1.1 (4)
N4—N3—C2—C18	1.1 (4)	C13—C12—C17—C16	−1.1 (4)
C1—N1—C3—C12	−82.3 (3)	C3—C12—C17—C16	180.0 (2)
N2—N1—C3—C12	95.4 (2)	C15—C16—C17—C12	−0.3 (4)
C1—N1—C3—C4	154.4 (2)	N2—C2—C18—C23	36.4 (3)
N2—N1—C3—C4	−27.9 (3)	N3—C2—C18—C23	−144.2 (2)
N1—C3—C4—C5	−59.6 (2)	N2—C2—C18—C19	−140.3 (2)
C12—C3—C4—C5	178.04 (19)	N3—C2—C18—C19	39.1 (3)
C3—C4—C5—O1	−28.0 (3)	C23—C18—C19—C20	0.1 (3)
C3—C4—C5—C6	153.21 (19)	C2—C18—C19—C20	176.8 (2)
O1—C5—C6—C11	−177.3 (2)	C18—C19—C20—C21	−0.3 (4)
C4—C5—C6—C11	1.4 (3)	C19—C20—C21—C22	0.4 (4)
O1—C5—C6—C7	1.7 (3)	C19—C20—C21—C24	−179.8 (2)
C4—C5—C6—C7	−179.6 (2)	C20—C21—C22—C23	−0.4 (4)
C11—C6—C7—C8	0.0 (4)	C24—C21—C22—C23	179.7 (2)
C5—C6—C7—C8	−179.1 (2)	C21—C22—C23—C18	0.3 (4)
C6—C7—C8—C9	2.0 (4)	C19—C18—C23—C22	−0.1 (3)
C7—C8—C9—C10	−2.3 (4)	C2—C18—C23—C22	−176.9 (2)
C8—C9—C10—C11	0.5 (4)		

### Hydrogen-bond geometry (Å, °)

**Table:**

*D*—H···*A*	*D*—H	H···*A*	*D*···*A*	*D*—H···*A*
N4—H4B···S1^i^	0.93 (3)	2.77 (3)	3.526 (3)	139 (2)

Symmetry codes: (i) −*x*, −*y*+1, −*z*.
